# Extracellular Vesicles in Cancer Diagnosis and Therapy: Advances, Challenges, and Prospects for Clinical Translation

**DOI:** 10.3390/ijms27052280

**Published:** 2026-02-28

**Authors:** Lingyu Kong, Guangyu Zhao, Xinwei Wu, Shuang Ma

**Affiliations:** 1 School of Sports, Shenyang Ligong University, Shenyang 110159, China; 2 School of Information Science and Engineering, Shenyang Ligong University, Shenyang 110159, China

**Keywords:** extracellular vesicles, liquid biopsy, cancer diagnosis, cancer therapy, exercise-related health

## Abstract

Extracellular vesicles (EVs) have emerged as promising tools for cancer diagnosis and therapy owing to their excellent biocompatibility, low immunogenicity, and ability to transport diverse bioactive molecules. This review summarizes recent advances in EVs research, focusing on isolation and detection technologies, their diagnostic and therapeutic applications in oncology, and the key challenges limiting clinical translation. Conventional EVs isolation methods, including ultracentrifugation, density-gradient centrifugation, and polymer-based precipitation, are discussed alongside emerging strategies such as immunoaffinity enrichment, microfluidic separation, lipid-mediated isolation, and thermophoretic enrichment, with comparative evaluation of their yield, purity, cost, and scalability. In cancer diagnosis, EV-associated biomolecules, such as miRNAs, mRNAs, proteins, and lncRNAs, show strong potential as liquid biopsy biomarkers for noninvasive early detection and dynamic disease monitoring. In therapeutic contexts, EVs serve as versatile carriers for gene molecules, chemotherapeutic agents, and small-molecule drugs, and can enhance immunotherapy and RNA-based treatments. Importantly, EVs released from metabolically active tissues, particularly skeletal muscle, contribute to systemic immune regulation and metabolic homeostasis, and their biogenesis and molecular cargo can be influenced by physical activity and exercise-related nutritional status. These insights highlight the need to integrate microengineering technologies, biomolecular profiling, standardized manufacturing systems, and lifestyle-related factors such as exercise and nutrition to accelerate the clinical translation of EV-based strategies in precision oncology and regenerative medicine.

## 1. Introduction

Cancer has one of the highest incidence and mortality rates globally. Owing to factors such as population aging, lifestyle changes, and environmental impacts, early screening, diagnosis, and treatment of cancer have become imperative [1]. However, many solid tumors often lack obvious clinical symptoms in the early stages. Conventional imaging examinations and tissue biopsies are insufficient for the timely detection of small lesions.

**Figure 1:**
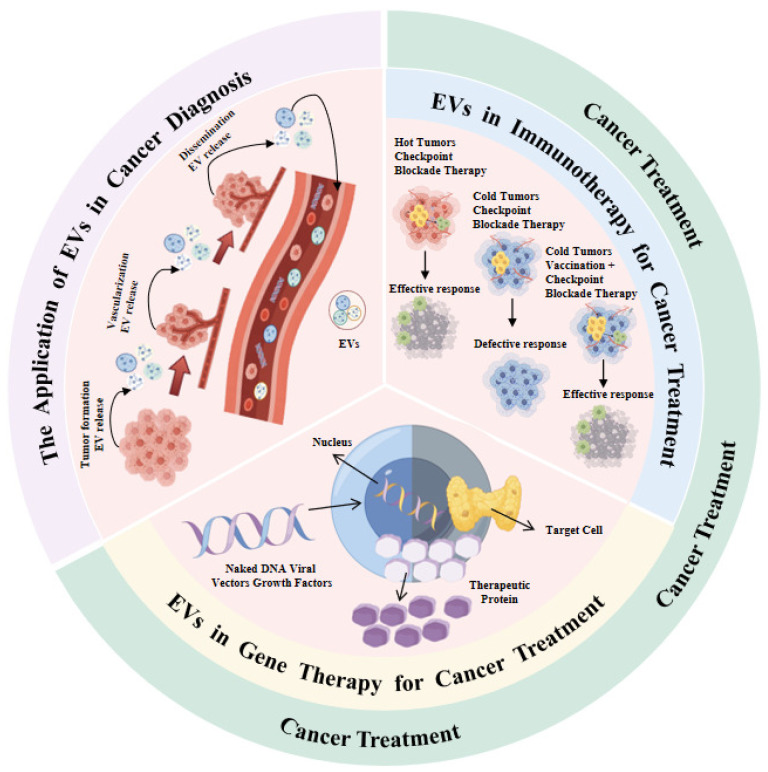
The multifaceted applications of EVs in cancer diagnosis and treatment. In cancer diagnosis, EVs enable noninvasive testing through the detection of tumor-derived molecular markers. In cancer therapy, EVs enhance treatment precision by optimizing immunotherapeutic efficacy (e.g., in “cold tumors”) and acting as efficient delivery vectors in gene therapy, facilitating the intracellular transport of therapeutic components (such as naked DNA, viral vectors, or growth factors) into target cells to drive the expression of anti-tumor therapeutic proteins [12].

Furthermore, traditional biopsies are highly invasive and associated with inherent risks, making it difficult to meet the requirements of real-time and dynamic monitoring [2]. In recent years, liquid biopsy technology has emerged as a novel strategy for the early diagnosis, prognostic evaluation, and therapeutic efficacy monitoring of cancers. This is achieved by detecting circulating tumor cells (CTCs), cell-free nucleic acids (cfDNA/ctDNA, cfRNA), and extracellular vesicles (EVs) in biological fluids, such as blood, urine, and other bodily secretions or excretions [3]. Among these components, EVs have considerable potential for clinical translation owing to their favorable stability, facile collection, capacity to shuttle a diverse array of biomolecules derived from parental cells (e.g., proteins, mRNA, miRNA, lncRNA, and circRNA), and relatively stable presence in bodily fluids. In the context of cancer diagnosis, EVs facilitate noninvasive and high-efficiency screening through the detection of their cargo molecular markers [4]. In cancer therapy, EVs not only function as a “booster” for immunotherapy. For example, when combined with vaccines in “cold tumors”, they remarkably enhance the response rate of immune checkpoint inhibition therapy and serve as vectors for gene therapy. By acting as natural nanocarriers, EVs enable the efficient intracellular delivery of genetic materials (such as naked DNA or viral vectors) and signaling molecules (like growth factors) into target cells, subsequently driving the expression and secretion of therapeutic proteins to exert potent anti-tumor effects (Figure 1) [5].

Beyond tumor-derived vesicles, EVs also act as systemic biological mediators released by metabolically active tissues, particularly skeletal muscle [6]. As the largest endocrine organ in the human body, skeletal muscle secretes a substantial number of EVs in response to physical activity. These skeletal muscle-derived EVs carry myokines, regulatory RNAs, and metabolic signaling molecules that participate in inter-organ communication, influencing immune regulation, inflammatory responses, and metabolic homeostasis [7]. Increasing evidence indicates that regular exercise, together with optimized exercise nutrition—such as adequate protein intake, balanced lipid consumption, and antioxidant-rich diets, can modulate the biogenesis, membrane composition, and molecular cargo of EVs [8]. This systemic regulatory role highlights EVs as a critical interface linking physical activity, nutritional status, and cancer-related biological processes, thereby providing a broader physiological context for EV-based cancer diagnosis and therapy.

Despite the inherent biocompatibility, low immunogenicity, and favorable targeting capabilities of EVs, their clinical translation is hindered by several critical challenges. These include the development of efficient methods for isolating high-purity EVs from complex biological samples (e.g., blood), identification and integration of multiple EV markers to establish detection panels with both high sensitivity and specificity, realization of large-scale production processes that ensure stable and consistent EV-based therapeutic formulations under good manufacturing practice(GMP) standards, and rigorous validation of their safety and efficacy through large-scale, multi-center clinical trials.

This study first provides an overview of the isolation and detection technologies for EVs, encompassing traditional methods such as ultracentrifugation (UC) and density gradient centrifugation, as well as emerging approaches, including immunoaffinity enrichment, microfluidic chips, thermophoretic enrichment, and lipid-mediated separation [9]. The advantages and disadvantages of these technologies in terms of purity, yield, operational convenience, and throughput are analyzed. Subsequently, we elaborate on the application value of EVs in cancer diagnosis, exploring the multi-marker combination detection strategy using prostate and pancreatic cancers as examples. It then summarizes the latest advances in EVs in fields including gene therapy, small-molecule drug delivery, immunotherapy, and RNA therapy [10]. Finally, an in-depth analysis of the key challenges faced in translating EVs into clinical practice is conducted, and we look forward to the future realization of the practical application of EVs in precise tumor diagnosis and treatment, which is anticipated through the collaborative innovation of engineering, biology, and clinical medicine [11].

## 2. Separation and Detection of EVs

### 2.1. Disadvantages of Traditional Separation Methods

The traditional isolation methods of EVs and their shortcomings are as follows: ultracentrifugation (UC) is time-consuming, requires expensive equipment, and yields products with low purity and throughput; Ultrafiltration (UF) is prone to membrane clogging and shear damage, making it difficult to remove impurities of the same size; Size exclusion chromatography (SEC) has relatively low efficiency and recovery rate, but its purity is relatively high; polymer precipitation is likely to introduce polymer contamination and non-specific precipitation, and commercial kits often obtain protein complexes rather than high-purity EVs when processing plasma, which limits clinical application; and the immunoaffinity method has high cost, excessive selectivity, and low recovery rate, so it is also not suitable for large-scale use [13]. Although the precipitation method offers relative convenience for processing cell culture media, it exhibits insufficient efficacy when applied to human samples and incurs high costs. Therefore, it is typically combined with techniques such as immunoaffinity to enhance the purity of isolated EVs [14].

### 2.2. Emerging Separation Methods

**Figure 2:**
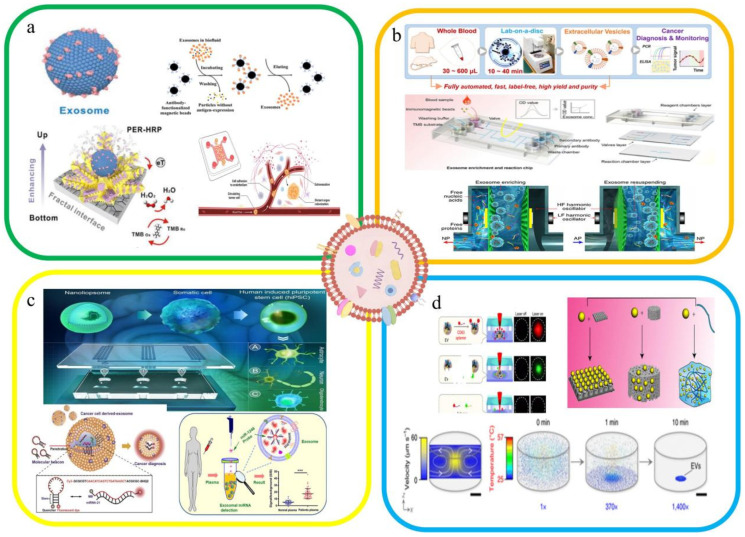
New techniques for EV isolation. (**a**) Immunoaffinity enrichment method [34,42,43]. (**b**) Separation based on physical features [44,45,46]. (**c**) Lipid-mediated separation [47,48]. (**d**) Thermophoresis enrichment [49,50].

To address the limitations of traditional EV isolation methods, including low purity, low yield, high cost, and potential damage to EVs, researchers have developed four novel enrichment technologies: immunoaffinity enrichment, physical feature-based separation, lipid-mediated separation, and thermophoretic enrichment [15]. The identification of EV-specific markers (e.g., Glypican-1 (GPC-1) and Epithelial cell adhesion molecule) has facilitated the precise isolation of EVs and their respective subpopulations [16]. The principles of traditional EV isolation methods are outlined as follows (Figure 2): UC separates particles based on their sedimentation velocity. It first precipitates denser non-EV particles, then collects EVs, and subsequently removes soluble impurities using an appropriate buffer solution. UF employs membranes with a specific molecular weight cutoff to retain EVs while allowing smaller proteins and particles to pass. SEC achieves separation by enabling particles of different sizes to elute at distinct rates through a porous matrix, with EVs collected in specific-elution fractions. Polymer precipitation modulates the solubility of EVs and soluble proteins, thereby enabling their recovery at relatively low centrifugation speeds [17]. In contrast, immunoaffinity enrichment leverages antibody-conjugated magnetic beads to selectively bind target EVs, followed by the separation of bound EVs from unbound impurities using a magnetic field [18]. This study summarizes the characteristics and potential applications of these novel EV isolation technologies (Table 1).

#### 2.2.1. Immune Affinity Enrichment

Owing to its operational convenience and high precision, the immunoaffinity separation approach has been employed in numerous studies for EV enrichment (Figure 2a). To achieve this objective, various technologies have been developed. For instance, Zhang et al. fabricated anti-CD81-functionalized microfluidic chips to isolate EVs from plasma samples [30]. Although these methods exhibit excellent performance in capturing EVs, the dissociation of the captured EVs remains problematic. The newly proposed on-demand EV chips and covalent chemistry-mediated EV click chips demonstrate promising potential for application in cancer detection [31]. Despite their high recovery rates and purity, these EV isolation technologies are associated with high costs and a reliance on specific markers. Concurrently, attention must be directed towards developing non-destructive EV release methods.

#### 2.2.2. Separation Based on Physical Characteristics

Microfluidic chips based on size exclusion have been employed to separate EVs from larger cellular debris, other membranous vesicles, and bubbles [32] (Figure 2b). Liu et al. developed a size-based EV separation chip, designated as the EV Total Isolation Chip, for comprehensive EV isolation. This chip can effectively extract EVs from cellular debris; however, membrane pore clogging remains a prominent challenge [33]. To address this issue, the system facilitates the passage of small particles (e.g., impurities) by switching between negative and positive air pressures while retaining larger EVs. Additionally, tangential flow filtration has been employed to mitigate the risk of membrane-pore clogging. Notably, the Exodisc developed by Sunkara et al. achieved a higher EV yield than conventional size-based isolation approaches [34]. In addition, deterministic lateral displacement achieves separation based on differences in particle size; however, this technology exhibits low throughput and is susceptible to the influence of vesicle density and mechanical rigidity of the particles.

#### 2.2.3. Lipid-Mediated Separation

The affinity-mediated capture of EV is enabled by the lipid molecules on the affinity-mediated EV capture (Figure 2c). To rapidly capture EVs from plasma samples, Wan et al. developed lipid-based nanoprobes that utilized biotin-labeled probes; notably, this approach shortened the separation time to 15 min [35]. Cholesterol-PEG1000 enhances the capture efficiency of EVs, whereas T cell membrane protein 4 enables the efficient capture of EVs enriched in phosphatidylserine. This lipid-mediated capture strategy leverages the interaction between specific lipid/protein molecules and EV surface components, offering a targeted approach for isolating EVs without relying on traditional size- or density-based separation principles [36]. In addition, Pang et al. utilized Fe_3_O_4_@TiO_2_ nanoparticles for EV capture, achieving a capture efficiency of up to 96.5% within five minutes. Notably, this integrated system also enables the simultaneous identification of target miRNAs from the isolated EVs, realizing the dual functions of rapid EV enrichment and downstream molecular detection, an advantage that streamlines the workflow for EV-based diagnostic applications [37,38]. These methods demonstrate the critical role of lipid molecules in the capture of EVs.

#### 2.2.4. Thermophoretic Enrichment

Thermophoresis refers to the migration of particles from a high-temperature region to a low-temperature region along a temperature gradient, with localized laser heating inducing this process [39]. To address the issue of purification procedures and lengthy separation, Liu et al. developed a highly sensitive thermophoretic technique for concentrating tumor-derived EVs. Using an adapter specific to the targeted tumor markers, rapid separation and enrichment of EVs from other components is achieved via thermophoresis, eliminating the need for prior isolation steps (Figure 2d). The accumulation of EVs generates amplified adaptive fluorescence signals, facilitating the analysis of EV surface biomarkers and detection of miRNAs [40]. To detect EVs in 1 μL of plasma, Tian et al. performed a similar analysis. This method provides a cost-effective and highly sensitive approach for liquid biopsies in patients with metastatic breast cancer [41].

## 3. Application of EVs in Cancer Diagnosis

EVs derived from cancer cells have emerged as potential biomarkers for cancer diagnosis [51]. These EVs carry a large array of biomolecules, including miRNAs, proteins, and nucleic acids, that can reflect the biological characteristics of tumors and provide valuable information for cancer diagnosis [52]. EVs can be isolated from bodily fluids, such as blood, urine, and saliva, and hold considerable potential for noninvasive cancer detection through liquid biopsy technology [53].

### 3.1. Diagnostic Potential of EVs

The unique properties of EVs endow them with notable advantages for cancer diagnosis. Cancer cells typically release a greater number of EVs than normal cells, and the biomolecules encapsulated within these EVs can indicate the origin and physiological state of the tumor [54]. For instance, in pancreatic ductal adenocarcinoma (PDAC), EVs contain specific miRNAs, such as miR-451a and miR-196a, and proteins such as GPC-1 [55]. Despite the profound clinical challenge that PDAC is typically diagnosed at advanced stages, recent breakthroughs have demonstrated the remarkable potential of EV-derived biomarkers for early detection. For instance, Halder et al. recently reported that serum exosomal ALPPL2 and THBS2 can discriminate patients with PDAC, importantly, including early-stage disease (Stages I and II), from individuals with non-cancerous pancreatic conditions and healthy controls with exceptional accuracy (AUC > 0.980) [56]. Additionally, EVs are enriched in various RNA species, including miRNAs, mRNAs, and lncRNAs, which play crucial roles in regulating cancer-related metabolic processes and cellular functions [57]. For example, the expression level of miR-451a in EVs isolated from serum is associated with PDAC staging, making it a valuable biomarker for both cancer diagnosis and prognosis.

### 3.2. Advantages of EVs in Liquid Biopsy

**Figure 3:**
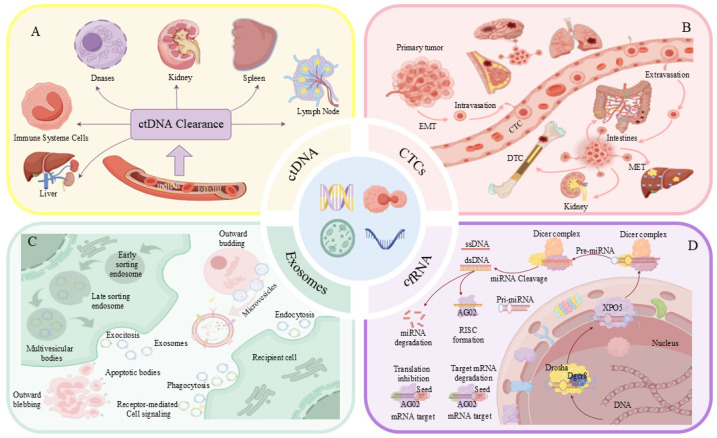
Various liquid biopsy techniques play crucial roles in early cancer screening, treatment monitoring, and recurrence prediction. (**A**) Identification of mutations and genetic alterations characteristic of cancer through the detection of ctDNA fragments in the bloodstream [59]. (**B**) Detection of CTCs in the blood provides real-time dynamic insights into tumor status [56]. (**C**) Prediction of drug response and investigation of the tumor microenvironment by analyzing informational molecules, such as DNA, RNA, and proteins, encapsulated within EVs [72]. (**D**) Detection of circulating cfRNA in the blood, which aids in assessing tumor activity and gene expression [59].

Liquid biopsy, which leverages components such as EVs, offers several advantages over traditional tissue biopsy. It is noninvasive, thereby reducing the risk of pain, infection, and tissue damage associated with conventional tissue sampling. Additionally, liquid biopsy enables real-time monitoring of metastasis, cancer progression, and treatment response [58]. Liquid biopsy can provide dynamic information about tumor status by detecting ctDNA, CTCs, and EVs in the blood. Although CTCs and ctDNA can offer insights into gene mutations and tumor heterogeneity, EVs have unique advantages in terms of stability; they are relatively stable in the bloodstream and can carry a variety of biomolecules derived from tumor cells, including RNA, proteins, and metabolites. Different liquid biopsy technologies, such as those targeting ctDNA, CTCs, EVs, and cfRNA, play crucial roles in cancer screening, treatment monitoring, and recurrence prediction (Figure 3) [59]. The characteristics, limitations, and potential clinical applications of different liquid biopsy technologies for cancer diagnosis are summarized in Table 2.

### 3.3. Clinical Applications of EVs in Cancer Diagnosis

EVs are gradually proving to play important roles as diagnostic tools in various types of cancer, including pancreatic cancer, lung cancer, and glioma. The biomolecules encapsulated within EVs provide valuable information regarding tumor characteristics, gene mutations, protein expression profiles, and the tumor microenvironment [73]. For example, in PDAC, EVs contain specific miRNAs that hold considerable diagnostic value for the early stages of the disease. Additionally, by analyzing proteins (e.g., CD63, CD9, and TSG101) in EVs, it is feasible to distinguish cancer cells from normal ones.

### 3.4. Exercise and Nutritional Status as Confounders and Modulators of EV-Based Liquid Biopsy

Growing evidence suggests that physical activity and nutritional status can reshape the landscape of circulating EVs, including their concentration, size distribution, and cargo composition. This introduces a clinically relevant confounding factor for EV-based liquid biopsy, as exercise-induced EV release, particularly from skeletal muscle and immune cells, may transiently elevate total EV counts and alter the signatures of inflammatory or stress-related cargoes [74]. As a result, EV-derived biomarkers measured in plasma/serum may be influenced by pre-analytical factors associated with lifestyle, which could potentially skew diagnostic readouts and contribute to inter-individual and inter-center variability. Importantly, these effects are not merely theoretical: acute exercise can induce rapid systemic alterations in cytokines, metabolic mediators, and immune cell trafficking, changes that may be reflected in EV cargo profiles and could obscure tumor-associated signals in the absence of standardized sampling protocols [75].

From a practical implementation perspective, EV-based diagnostics may therefore benefit from incorporating lifestyle-aware standardization strategies. These include harmonizing patient preparation (e.g., fasting state, exercise restriction within a defined time window before blood draw), documenting recent physical activity and dietary intake as part of clinical metadata, and evaluating the stability of candidate biomarker panels against exercise/nutrition perturbations in pilot cohorts [76]. In addition, marker selection should prioritize tumor-enriched signals with demonstrated robustness across diverse physiological states, while panel-based approaches can incorporate normalization features (e.g., analyte ratios or multi-analyte signatures) to mitigate sensitivity to global EV fluctuations [77]. Collectively, treating exercise and nutrition as measurable covariates rather than uncontrolled noise may improve the reproducibility and clinical interpretability of EV-based liquid biopsy assays.

### 3.5. Practical Framework for Multi-Marker Panel Selection and Standardization

A practical approach to addressing EV heterogeneity and the limitations of single-marker strategies is to transition from “single-marker claims” to clinically validated multi-analyte panels paired with standardized workflows. In panel construction, marker selection should be guided by three core principles: clinical intent (e.g., early detection, prognosis, or treatment monitoring), biological plausibility (encompassing tumor enrichment and mechanistic relevance to disease pathogenesis), and analytical feasibility (including detectability under realistic sample volumes and clinically acceptable turnaround times) [78]. Rather than relying on a single EV surface protein or a single miRNA, integrated panels can combine complementary layers, such as EV surface proteins for tissue-of-origin enrichment, RNA signatures reflecting tumor programs, and mutation-related signals where applicable. Importantly, the robustness of these panels should be explicitly tested against major sources of variability, including EV subtype heterogeneity, pre-analytical handling procedures, and physiological covariates.

For real-world implementation, standardization should encompass harmonized pre-analytical protocols (e.g., anticoagulant type, processing time, centrifugation parameters, storage temperature, and freeze–thaw limits), orthogonal EV characterization (including particle counts, size distribution, and contaminant assessment), and consistent analytical platforms across clinical centers [79]. Model development should adhere to a staged validation strategy, encompassing an internal training cohort, an independent validation cohort, and multicenter external validation with pre-specified performance metrics. Threshold definition and normalization strategies are equally critical; for instance, panel scores can be constructed using weighted combinations or analyte ratios to mitigate global EV fluctuations, while internal controls can be integrated to monitor RNA extraction and detection efficiency [80]. Finally, given that EV composition and tumor dependencies vary across cancer types, the transferability of multi-analyte panels should not be assumed; cancer-specific optimization and stratified evaluation are therefore necessary to preclude overgeneralization.

## 4. Application of EVs in Cancer Therapy

With the deepening of research on EVs, their potential in cancer treatment is gradually emerging, making them an important component of novel cancer therapeutic strategies. The main therapeutic applications of EVs in cancer treatment include gene delivery, drug delivery, immunotherapy, and RNA therapy [81]. To further enhance the understanding of these therapeutic strategies, this article summarizes the diverse applications of EVs in cancer treatment and their comparative analysis across different clinical stages (Table 3).

### 4.1. Application of Gene Delivery Technologies in the Treatment of Cancer and Genetic Diseases

As an emerging therapeutic modality, gene therapy achieves therapeutic effects by repairing or modifying the genetic material in a patient’s genome. Gene delivery technology is a key step in enabling gene therapy, which primarily involves introducing exogenous genes into target cells to repair defective genes or introduce new functional genes [96]. Common gene delivery technologies include viral vectors, non-viral vectors, EVs, and physical methods.

Viral vectors are among the most widely used technologies for gene delivery. Exogenous genes can be efficiently delivered to target cells using viral vectors, such as adenoviruses and lentiviruses. Owing to their high delivery efficiency and strong targeting capability, viral vectors are valuable in the treatment of cancer and genetic diseases. However, viral vectors may trigger immune responses, which can lead to rejection by the host immune system, thereby compromising their therapeutic efficacy [97]. Additionally, viral vectors incur relatively high production costs, and multiple administrations may be required to sustain their therapeutic effects.

Non-viral vectors deliver genes using materials such as liposomes and polymers. They offer advantages, including a low risk of immune response and low production costs, making them suitable for treatments that require frequent administration [98]. Nevertheless, non-viral vectors exhibit relatively low gene delivery efficiency and face challenges related to stability and bioavailability. Therefore, further optimization is required to facilitate their effective clinical application.

As natural nanoparticles, EVs have unique advantages in gene delivery owing to their high biocompatibility, low immunogenicity, and strong targeting capability. EVs can effectively fuse with the membranes of target cells and have demonstrated potential in fields such as cancer treatment and neurodegenerative disease therapy [99]. However, the production and purification processes of EVs are relatively complex and costly, limiting their large-scale application. Currently, EV-mediated gene delivery technology is primarily in preclinical and early phase clinical trial stages [100].

Physical methods, such as electroporation and microinjection, directly introduce genes into cells via physical means. This approach is characterized by its simplicity and high universality, as it is applicable to various cell types. Despite these merits, physical methods exhibit low delivery efficiency and may cause damage to cells, limiting their wide application in clinical practice [101]. However, physical methods remain important in basic research, particularly in studies involving cell models of cancer and genetic diseases.

### 4.2. Application of EVs as Drug Carriers in Chemotherapy and Small-Molecule Drug Delivery

The application of EVs as drug carriers has demonstrated considerable advantages in the delivery of chemotherapeutic and small-molecule drugs. As natural vesicles secreted by cells, EVs can carry biomolecules such as DNA, RNA, and proteins. They also possess inherent cell membrane fusion properties, which enable the efficient delivery of drugs to target cells [102]. In terms of chemotherapeutic drug delivery, EVs, as natural drug carriers, can bind effectively to the membranes of target cells and deliver drugs into the cell interior. This property enables them to transport drugs precisely to tumor cells, boosting drug levels at the tumor site while reducing toxicity to surrounding normal tissues [103].

EVs also have important applications in small-molecule drug delivery. Small-molecule drugs typically exhibit low bioavailability and short in vivo half-lives, limiting their therapeutic effects to a certain extent. As natural carriers, EVs can protect small-molecule drugs from degradation, improve their stability, and extend their in vivo circulation time, thereby enhancing their therapeutic efficacy. The biomembrane structure of EVs can effectively prevent drugs from being degraded or inactivated in the bloodstream, preserving their therapeutic activity; this is of great significance, especially for diseases requiring long-term treatment, such as cancer and neurodegenerative diseases [103]. Thus, EVs have emerged as ideal carriers for small-molecule drugs with substantial therapeutic potential [104].

### 4.3. Application of EVs in Cancer Immunotherapy

EVs have gradually attracted attention in cancer immunotherapy because they can both facilitate tumor immune escape and activate anti-tumor immune responses. Tumor-derived EVs are enriched in immune-activating factors such as tumor-specific antigens, heat shock proteins, and MHC I/II–peptide complexes. These molecules promote the uptake of antigens by dendritic cells (DCs) and activate T cells, thereby inducing tumor cell apoptosis [72]. EVs can stimulate DCs to secrete cytokines and activate NK cells, thereby further enhancing the attack on tumor cells.

However, tumor-derived EVs also carry immunosuppressive factors such as PD-L1, CD73, TGF-β, and IL-10. These factors enable tumors to escape immune surveillance by inhibiting T cell proliferation and function. Additionally, EVs can promote the activation of regulatory T cells, which further suppresses the functions of effector T cells and natural killer (NK) cells. They can also induce the proliferation of myeloid-derived suppressor cells (MDSCs), thereby weakening the effector function of T cells [105].

To address these challenges, researchers are exploring the combined application of EVs with immunotherapy, such as the combination of EVs and immune checkpoint inhibitors (e.g., anti-PD-1/PD-L1 antibodies) or utilizing EVs as delivery carriers for cancer vaccines. Furthermore, EVs can support chimeric antigen receptor T-cell (CAR-T) therapy by enhancing the capacity of CAR-T cells to recognize and eliminate tumor cells. Additionally, by developing inhibitors or blocking antibodies that target immunosuppressive molecules on the EV surface (such as PD-L1 and CD73), the function of T cells can be restored, and the efficacy of immunotherapy can be further improved [106].

**Figure 4:**
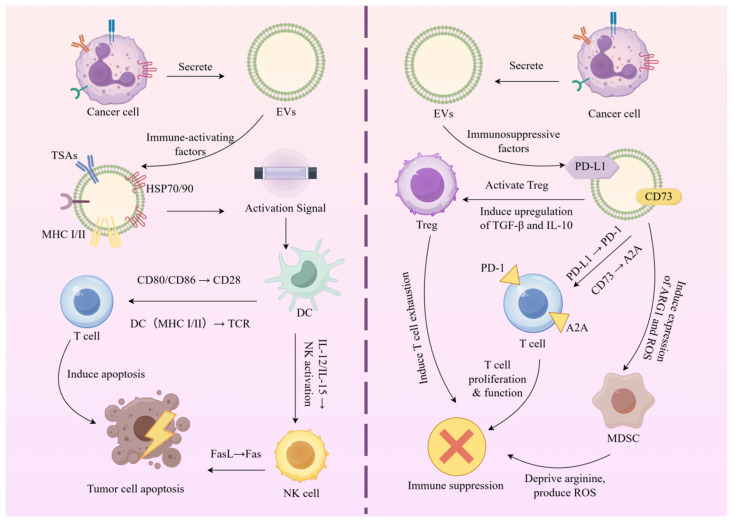
Dual Mechanisms of EVs in Cancer Immunotherapy [107].

As shown in the left panel of Figure 4, EVs secreted by cancer cells carry immune-activating factors, such as tumor-specific antigens, heat shock proteins, and MHC I/II molecules, which activate NK cells, DCs, and T cells, thereby inducing anti-tumor immune responses [107]. The right panel demonstrates that cancer cell-derived EVs carry immunosuppressive factors, such as PD-L1 and CD73. By binding to receptors on immune cells, these factors inhibit T cell function and facilitate tumor immune escape. Additionally, EVs may further exacerbate the formation of an immunosuppressive microenvironment by activating MDSCs and T cells, thereby providing enhanced protection against immune attacks [7].

### 4.4. Application of RNA Delivery Mediated by EVs in Cancer Therapy

RNA-based gene therapy strategies, including small interfering RNA (siRNA), microRNA (miRNA) mimics, and messenger RNA (mRNA), have emerged as pivotal tools for modulating the expression of cancer-associated genes [10]. However, the clinical translation of RNA therapy remains hampered by multiple limitations, including rapid in vivo degradation, low cellular uptake efficiency, and potential systemic toxicity. In this context, EVs have garnered extensive attention owing to their inherent properties as natural nanocarriers. EVs exhibit excellent biocompatibility, low immunogenicity, and an innate capacity to mediate intercellular communication, rendering them highly promising platforms for RNA delivery.

EVs have been widely employed for the delivery of siRNAs targeting oncogenic driver genes, including KRAS, PLK1, and MYC. Of particular note, siRNA-loaded EVs (termed iExosomes) have entered early-phase clinical evaluation for therapeutic development in patients with PDAC [108]. This progress represents a critical milestone in the translation of EV-mediated RNA delivery from basic research to clinical application. However, current publicly available data are largely limited to safety profiles and preliminary observations of biological activity. Evidence regarding objective response rates, long-term survival benefits, and comparisons with standard therapies remains relatively scarce, indicating that therapeutic efficacy requires further validation in large-scale controlled clinical trials.

Compared to synthetic lipid nanoparticles (LNPs), EVs are generally considered to exhibit superior biocompatibility and lower immunogenicity. Nevertheless, this advantage appears inconsistent across different research models. The LNP platform has achieved clinical validation in applications including mRNA vaccines, with well-established manufacturing processes, dose specifications, and quality control (QC) systems. In contrast, EVs are characterized by diverse origins, complex compositions, and greater batch-to-batch variability [109]. Following systemic administration, EVs are often rapidly cleared by the mononuclear phagocyte system and accumulate predominantly in organs such as the liver and spleen, thereby limiting the effective dose delivered to tumor tissues. Accordingly, relying solely on their “natural origin” to guarantee safety and efficacy is insufficient, and further systematic pharmacokinetic evaluations and in vivo biodistribution studies are required to validate their translational potential.

Currently, prevalent siRNA loading strategies include electroporation, lipid-assisted transfection, and genetic engineering of donor cells to enable endogenous siRNA packaging. Furthermore, surface engineering modifications (e.g., fusion peptide RVG or ligand conjugation) can further enhance the tumor-targeting delivery capacity of EVs [110]. However, substantial variations in efficiency and reproducibility exist among different loading methods. Electroporation may induce RNA aggregation or membrane damage, impairing vesicle integrity; while endogenous loading is relatively gentle, its RNA packaging efficiency is governed by the physiological state of donor cells, making precise control of payload copy number per EV challenging. Critically, unified standards for defining functional doses remain lacking. The commonly used metrics of “particle number” or “total RNA yield” fail to accurately reflect the actual number of functional RNA molecules that enter target cells and elicit biological effects, presenting substantial challenges for clinical dose optimization and therapeutic efficacy prediction.

Numerous preclinical studies have validated that EV-mediated siRNA delivery enables efficient gene silencing and suppresses tumor growth in models of pancreatic cancer and glioblastoma. However, siRNA stability continues to represent a major challenge during clinical translation [57]. During storage and freeze–thaw cycles, RNA may undergo partial degradation. Even if total RNA quantification shows no substantial reduction, its functional activity can still be compromised. Furthermore, RNA-loaded EVs and their surface components may still trigger endogenous immune recognition pathways, such as Toll-like receptors or cytoplasmic RNA sensors, thereby inducing the release of inflammatory mediators. In certain patient populations, such innate immune activation may carry potential safety risks that require evaluation via systematic immune monitoring.

For miRNA delivery, tumor-suppressive miRNAs (e.g., miR-34a and miR-16) have been delivered via EVs to restore dysregulated gene regulatory networks in cancer. Studies have demonstrated that EV-mediated miRNA delivery can induce apoptosis, inhibit epithelial-mesenchymal transition, and reduce metastasis in hepatocellular carcinoma, breast cancer, and colorectal cancer models [111]. However, the pleiotropic effects of miRNAs, an inherent feature of their multi-target gene regulation, represent both a potential advantage and a source of risk. Substantial differences in gene dependency exist across tumor types and even among molecular subtypes within the same tumor, which may lead to inconsistent efficacy or unintended signaling interference. Thus, careful target selection and rigorous patient stratification are critical for robust clinical trial design.

In addition to gene-silencing strategies, EVs have also been employed to deliver therapeutic mRNAs encoding tumor-suppressive proteins (e.g., p53), suicide genes, or immunostimulatory cytokines. Preclinical studies have demonstrated that EV-delivered mRNAs enable functional protein expression in target cells and suppress tumor progression [112]. However, similar to siRNAs, mRNA molecules are longer and structurally more complex, rendering them more sensitive to storage conditions and loading efficiency. Their translation efficiency is strongly influenced by cell type, intracellular trafficking pathways, and the immune microenvironment. Furthermore, large-scale production of engineered donor cells, stable expression of specific mRNAs, and maintenance of consistent EV quality remain technically challenging and cost-prohibitive under GMP conditions.

Although EV-mediated RNA delivery theoretically offers biocompatibility advantages and intrinsic tissue tropism conferred by its native membrane structure, its clinical success relies on the synergistic optimization of multiple factors, including payload stability, dose standardization, immune safety evaluation, large-scale production capacity, and systematic comparisons with established delivery platforms (e.g., LNPs and viral vectors) [72]. In future studies, integrating engineering optimization with rigorous clinical validation, while incorporating immune monitoring, pharmacokinetic profiling, and biomarker-based stratification design at an early stage, will facilitate a more accurate assessment of the practical feasibility and risk-benefit ratio of EV-mediated RNA therapy for cancer treatment.

**Figure 5:**
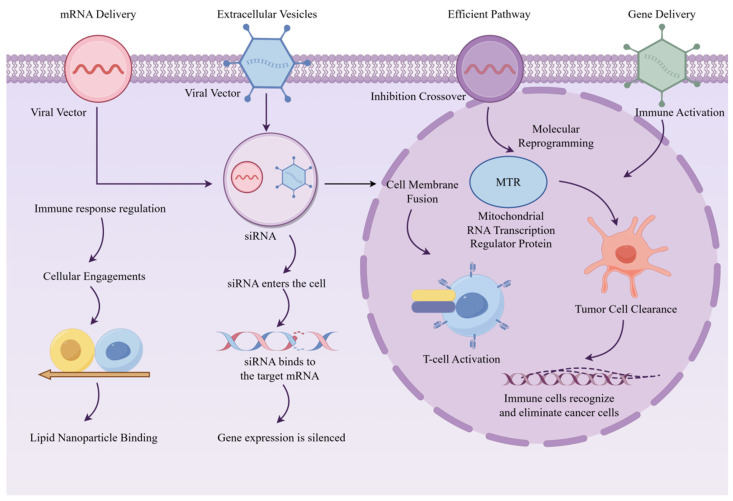
Schematic diagram of the key mechanisms of RNA therapy and its immune effect framework associated with delivery systems. The diagram summarizes the core intracellular events underlying the action of RNA therapeutics, including siRNA-mediated gene silencing and mRNA-mediated protein expression or molecular reprogramming. It further highlights that delivery systems (such as viral vectors, LNPs, and membrane-based nanocarriers including EVs) can modulate immune activation and T-cell responses by influencing cellular internalization and intracellular trafficking pathways, thereby shaping the efficiency of antitumor clearance [112].

To facilitate mechanistic understanding of the relationships among cellular entry pathways, RNA functional execution, and immune effects across different RNA delivery systems, Figure 5 provides a conceptual overview of canonical RNA delivery routes and their immunomodulatory outcomes. Collectively, the core functional modes of RNA therapies include the following: upon cytoplasmic entry, siRNAs hybridize with target mRNAs and mediate gene silencing; similarly, after entering the cytoplasm, mRNAs are translated into functional proteins to achieve molecular reprogramming or induce tumor cell death; in addition, delivery systems themselves and the cellular responses they elicit can further trigger immune activation and modulate T cell responses, thereby defining the magnitude and durability of antitumor immunity. Within this framework, as naturally derived membranous nanocarriers, EVs may improve the efficiency of functional RNA delivery via favorable intracellular trafficking following membrane fusion or endocytosis, and under certain conditions reduce the risk of nonspecific innate immune activation. Nonetheless, challenges, including biased in vivo distribution toward the liver and spleen, as well as inherent heterogeneity, can still compromise the effective dose delivered to tumor tissues. Thus, evaluating EV delivery pathways and immune effects within a unified mechanistic framework helps clarify inconsistencies in efficacy and safety observed across studies and provides rational guidance for further engineering optimization.

## 5. Translational Bottlenecks and Clinical Implementation Framework for EV-Based Cancer Therapies

Despite rapid advances in EV engineering and promising antitumor efficacy demonstrated in multiple preclinical models, the clinical translation of EV-based cancer therapies remains relatively slow [113]. The major reasons are not confined to technical optimization at the laboratory level, but extend to real-world implementation barriers, including scalable manufacturing, batch consistency, potency definition, standardized QC, clinical feasibility (cost, time, and infrastructure), multicenter reproducibility, and regulatory classification. Therefore, a clinically oriented discussion should evaluate EV technologies not only by their mechanistic novelty or delivery efficiency in experimental systems, but also by whether they can be manufactured reproducibly under GMP conditions, quantified using validated potency assays, stored and transported stably, and implemented across multiple centers with harmonized workflows [36].

### 5.1. Scalable Manufacturing and GMP-Compliant Production

A fundamental bottleneck for clinical translation is the lack of mature, scalable, and standardized production pipelines capable of consistently generating EV products with reproducible yield and biological activity. EV production is highly dependent on donor cell type, culture conditions, passage number, medium formulation, and bioreactor parameters, meaning that minor upstream fluctuations can result in substantial downstream variability in vesicle composition and function [114]. Traditional ultracentrifugation remains widely used in research laboratories but is labor-intensive, time-consuming, low-throughput, and difficult to scale. Furthermore, high gravitational forces and repeated centrifugation steps may compromise vesicle integrity and lead to co-isolation of contaminants. Scalable alternatives such as tangential flow filtration, size-exclusion chromatography, and other chromatography-based approaches can improve manufacturability, yet their robustness, cost-effectiveness, and contamination control still require systematic validation under GMP conditions. In practical clinical manufacturing, key requirements include establishing standardized donor cell banking and release criteria, adopting serum-free or chemically defined culture systems to minimize batch impurities, implementing closed and automated production workflows to reduce contamination risks, and defining reproducible purification processes that balance yield and purity [115]. Importantly, the cost and infrastructure burden of EV production can be substantial compared with certain synthetic nanocarriers. Without scalable bioprocessing and automation, EV therapeutics may remain restricted to small-scale or highly selected indications rather than achieving widespread clinical implementation.

### 5.2. Cargo Loading Efficiency and Functional Potency Assays

For therapeutic EVs, a major challenge is achieving stable and reproducible cargo loading while maintaining vesicle integrity and biological function. Current loading strategies, such as electroporation, sonication, membrane permeabilization, lipid-assisted loading, or endogenous loading via donor-cell engineering, often show substantial variability across laboratories and even across batches within the same laboratory [102]. Physical or chemical manipulation may also disrupt membrane structure, modify surface proteins, or promote aggregation, which can further affect biodistribution, safety, and therapeutic efficacy. Meanwhile, clinical translation is hindered by the lack of universally accepted potency assays and dose definition standards. Many studies rely on total RNA or protein quantification, but such measurements cannot reliably distinguish cargo genuinely encapsulated inside EVs from material adsorbed to the vesicle surface or co-isolated free nucleic acids and protein complexes [116]. For RNA therapeutics, key practical questions remain difficult to address using current routine assays, including how many functional siRNA or mRNA molecules are delivered per vesicle, what fraction reaches the cytosol of target cells, and how this quantity correlates with gene silencing or protein expression outcomes [116]. To support regulatory evaluation, EV products ultimately require orthogonal characterization and potency testing that correlate physical attributes (size distribution, concentration, purity, marker profile) with functional readouts (cell-based activity assays reflecting the mechanism of action), thereby enabling consistent batch release and clinically meaningful dose selection.

### 5.3. Biological Heterogeneity and QC Standardization

EV populations are inherently heterogeneous, comprising multiple vesicle subtypes with overlapping size ranges and partially shared markers, which complicates their identity definition and comparability. This heterogeneity impacts both diagnostic and therapeutic applications: in diagnostics, it can dilute disease-relevant signals, while in therapeutics, it may introduce functional variability and unpredictable immune interactions [117]. Although advanced analytical methods, including high-resolution flow cytometry, cryo-electron microscopy, and multiomics profiling, have improved EV characterization, no universally accepted consensus yet exists regarding identity, purity, and potency criteria for routine clinical use [118]. In real-world settings, QC should not rely on single metrics, but rather on a standardized panel encompassing vesicle concentration and size distribution, marker expression profiles, assessment of major contaminant classes (e.g., lipoproteins, protein aggregates, residual nucleic acids), sterility and endotoxin testing, as well as stability testing under clinically relevant storage and transport conditions [119]. Another practical limitation is the lack of widely accepted reference materials and harmonized calibration standards, which compromises inter-laboratory comparability and complicates the implementation of multicenter trials. Establishing standardized QC workflows and reference preparations will be essential to reducing center-to-center variability and supporting the regulatory acceptance of EV products.

### 5.4. Biodistribution, Targeting Efficiency, and Safety Considerations

While EVs are often attributed with intrinsic targeting or tissue tropism, systemic administration frequently leads to their predominant accumulation in clearance organs such as the liver and spleen, limiting the effective dose delivered to tumor tissues [120]. Rapid uptake by the mononuclear phagocyte system and nonspecific biodistribution can reduce the therapeutic index and increase off-target exposure. Engineering strategies, such as ligand conjugation, peptide display, or membrane remodeling, have shown promise in improving tumor tropism, yet their performance is often context-dependent and requires rigorous pharmacokinetic and biodistribution validation beyond small-animal models. Safety evaluation is equally critical [121]. Although EVs are frequently considered low-immunogenic, immune risks depend strongly on donor cell source, purification rigor, surface composition, and loaded cargo, and may include unintended innate immune activation, complement activation, procoagulant effects, or adverse cytokine responses. For nucleic-acid payloads, additional risks include off-target gene effects, variable intracellular trafficking, and durability of gene modulation [122]. Moreover, when EVs are combined with checkpoint blockade, vaccines, or cellular therapies, immunological interactions become more complex and may modulate both efficacy and safety. Therefore, long-term toxicology and immunogenicity studies, stability assessments including freeze–thaw sensitivity, and careful evaluation of tumor-promoting versus tumor-suppressing effects are essential before widespread clinical implementation.

### 5.5. Regulatory Classification and Clinical Trial Design

EV therapeutics occupy a hybrid regulatory space with characteristics of biologics, cell-derived products, and nanomedicines, and this complexity influences product definition, release specifications, and post-treatment monitoring requirements [120]. From a translational perspective, a feasible development pathway requires not only demonstrating preclinical efficacy but also establishing a robust manufacturing and control strategy, validated potency assays, and clinically actionable endpoints. Clinically, early-phase studies should prioritize dose-escalation safety evaluation, pharmacokinetic and biodistribution profiling, and mechanism-linked biomarkers that reflect target engagement and biological activity [123]. For therapeutic EVs, trial design must also account for patient heterogeneity, tumor-type variability, prior treatment exposure, and combination regimens that may influence immune responses and toxicity profiles. For diagnostic EV assays, multicenter validation is particularly challenging because variability can be introduced at every step, including sample collection timing, anticoagulant selection, processing delay, storage temperature, freeze–thaw cycles, differences in isolation protocols, and instrument calibration parameters [124]. Without strict harmonization of these factors, reproducibility may be inadequate to support regulatory approval or clinical adoption. Therefore, multicenter-ready workflows should incorporate standardized pre-analytical protocols, shared QC metrics, cross-laboratory proficiency testing, and predefined statistical plans for inter-site performance evaluation.

### 5.6. Future Directions and Cross-Disciplinary Integration

Future progress toward clinical translation will depend on integrating advances in bioengineering, manufacturing science, analytical chemistry, oncology, immunology, and regulatory science [114]. Clinically feasible EV products will likely require automated and closed-system production platforms, improved purification strategies that are scalable and contamination-resistant, and standardized release testing that links vesicle attributes to functional potency [125]. In parallel, diagnostics will benefit from rationally designed multi-analyte panels that account for EV heterogeneity and are validated in large, well-characterized cohorts using harmonized protocols. Engineering innovations that reduce nonspecific clearance and enhance tumor delivery, combined with rigorous safety monitoring frameworks, will be critical for improving the therapeutic index [126]. Finally, establishing reference materials, adopting consensus guidelines for characterization and reporting, and implementing multicenter harmonization strategies will collectively reduce variability and accelerate regulatory translation. With coordinated interdisciplinary efforts, EV-based platforms may advance from promising experimental modalities into reliable, safe, and clinically actionable tools for precision oncology.

## 6. Conclusions and Prospects

EVs are released into the extracellular milieu through multiple biogenesis pathways, including the multivesicular body route, and naturally carry diverse biomolecules such as RNAs, proteins, and lipids. This intrinsic cargo diversity underpins their central roles in intercellular communication and signal transduction, and provides a strong biological rationale for their use in cancer diagnostics and therapeutic delivery systems [103].

From a methodological perspective, EV isolation and purification remain a decisive factor governing downstream analytical reliability and clinical feasibility. Conventional approaches such as ultracentrifugation, density gradient centrifugation, and affinity capture continue to serve as widely used foundations, yet they are constrained by limitations including time consumption, sample loss, and potential vesicle damage. Meanwhile, emerging technologies, such as microfluidic platforms and alternating current-based separation, offer advantages in processing speed, specificity, and suitability for small-volume samples, although their scalability and robustness still require further validation. Overall, different techniques represent distinct trade-offs across key parameters, including sample input, operational complexity, throughput, and specificity, highlighting the importance of method selection based on intended clinical scenarios [127].

In oncology, EV-associated biomolecules (e.g., proteins, miRNAs, and tumor-derived DNA alterations) have demonstrated substantial promise as minimally invasive biomarkers for early detection, stratification, and prognosis in multiple cancer types, including prostate and pancreatic cancers [104]. However, the heterogeneity of EV populations and the limited performance of single-marker strategies underscore the need for validated multi-analyte panels and standardized workflows. In therapeutics, EVs show notable potential as carriers for drug and nucleic-acid delivery due to their biocompatibility and relatively low immunogenicity, yet key technical barriers, particularly loading efficiency, targeting precision, and safety control, remain to be systematically addressed before broad clinical implementation [72].

Several translational bottlenecks currently limit clinical adoption. High-purity and homogeneous EV preparation from complex biofluids is still challenging; robust diagnostic performance often requires multi-marker combinations rather than single EV markers; and therapeutic EV products must comply with stringent GMP manufacturing and quality standards to ensure batch consistency, safety, and efficacy. In addition, further advances in detection technologies are needed to simultaneously improve sensitivity, specificity, and throughput under clinically realistic constraints [105].

Beyond conventional oncologic frameworks, skeletal muscle-derived EVs are increasingly recognized as systemic regulators connecting physical activity, metabolic status, and immune function. Exercise and exercise nutrition may modulate EV biogenesis and molecular composition, suggesting that lifestyle-related factors could influence EV-based diagnostic signals and potentially affect therapeutic responsiveness. Integrating exercise science, skeletal muscle biology, and nutritional research with oncology and bioengineering may therefore support more comprehensive strategies for cancer prevention, adjunctive therapy, and long-term health management.

Ultimately, successful translation of EVs from preclinical research to clinical practice will require coordinated advances across engineering, biology, and clinical medicine. Priority directions include continuous optimization of isolation and analytical platforms, discovery and validation of reliable EV-associated biomarkers, establishment of standardized manufacturing and quality-control systems, and confirmation of clinical value through large-scale, multi-center clinical trials [106].

## Figures and Tables

**Table 1:** EV Isolation Techniques and Applications.

Techniques	Principle	Time Required	Sample Types	Sample Volume	Purity	Ease of Use	Cost	Application Potential (Level)	Ref.
**Immunoaffinity** **Enrichment**	Enriches and separates target molecules via specific binding of antibodies to target molecules and using solid matrices	1–3 h	Serum, plasma, urine, saliva, etc.	1–10 mL	High (depends on antibody specificity)	Moderate	High	High (suitable for high-throughput screening and diagnostic applications)	[19,20,21]
**Physical Feature-based separation**	Separates target molecules based on physical characteristics (e.g., size, density, shape, or charge)	1–4 h	Serum, plasma, cell culture supernatant, etc.	5–20 mL	Moderate to High (depends on separation method)	Moderate	Medium	Medium (suitable for large-scale separation but with low efficiency)	[22,23]
**Lipid Mediated-Separation**	Separates target molecules by utilizing the affinity of lipids through the interaction between lipid molecules and target molecules	2–6 h	Serum, plasma, cell culture supernatant, etc.	5–20 mL	High (depends on lipid affinity)	Moderate	Medium to High	High (suitable for fine separation and exhibits high affinity for EVs)	[24,25,26]
**Thermophoretic Enrichment**	Separates particles of different sizes or densities by inducing thermophoretic effect via temperature gradient	1–3 h	Serum, plasma, cell culture supernatant, etc.	1–10 mL	High (depends on particle size and density)	Moderate	Medium to High	Medium (suitable for small-scale separation and requires further optimization)	[27,28,29]

**Table 2:** Comparison of Different Liquid Biopsy Technologies for Cancer Diagnosis.

Technology	Detection Methods	Sample Types	Sample Volume	Sensitivity (%)	Specificity (%)	Features & Advantages	Limitations	Turnaround Time (Cost/Proposed Cost)	Ref.
**ctDNA analysis**	PCR, Next-generation sequencing (NGS), Digital PCR	Serum, Plasma, Urine	5–20 mL	70–90%	85–95%	High sensitivity, suitable for early cancer detection and monitoring of treatment response.	Complex sample processing, susceptible to background DNA contamination, high detection cost	1–3 days (High cost, dependent on sequencing platform)	[60,61]
**CTCs detection**	Microfluidic Technology, Immunomagnetic Bead Separation, Flow Cytometry	Serum, Plasma	5–20 mL	60–85%	85–95%	Enables real-time monitoring of cancer progression and evaluation of treatment efficacy	Low CTCs concentration, complex separation process, potential false negatives	1–5 days (Medium to high cost, dependent on technology platform)	[62,63]
**EVs detection**	Ultracentrifugation, Immunoaffinity Enrichment, Transmission Electron Microscopy (TEM), Nanoparticle Tracking Analysis (NTA)	Serum, Plasma, Urine, Saliva	1–20 mL	70–90%	80–95%	Non-invasive sampling, capable of detecting multiple biomarkers, with high specificity	Complex separation steps, limited purification efficiency, potential need for additional purification steps	1–3 days (Medium to high cost, dependent on separation and detection methods)	[64,65]
**Free RNA detection**	PCR, RT-PCR, Next-generation Sequencing (NGS)	Serum, Plasma, Urine, Saliva	1–10 mL	70–85%	80–95%	Capable of detecting free RNA biomarkers associated with early-stage cancer	Complex sample processing, susceptible to interference from other RNAs in plasma or serum	1–3 days (Medium cost, dependent on the detection method used)	[66,67]
**Protein markers**	Immunoassays (ELISA, Western Blot), Mass Spectrometry, Immunohistochemistry (IHC), Flow Cytometry	Serum, Plasma, Urine, Saliva	1–10 ml	60–95%	70–95%	Applicable for detecting specific cancer markers, with high clinical utility	Sensitivity may be limited by marker concentration, potential need for multiplex detection	1–3 days (Medium to high cost, dependent on the method used)	[68,69]
**Metabolite detection**	Nuclear Magnetic Resonance (NMR), Gas Chromatography-Mass Spectrometry (GC-MS), Liquid Chromatography-Mass Spectrometry (LC-MS)	Serum, Plasma, Urine, Saliva	1–5 mL	70–90%	80–95%	Enables broad metabolite detection, facilitating the discovery of potential biomarkers	Complex sample preparation, high instrument cost, difficult data analysis	1–3 days (Medium to high cost, dependent on the detection platform used)	[70,71]

**Table 3:** Overview of engineered EV modalities for cancer therapy.

EV Therapeutic Modality	Functional Component	Loading/Engineering Strategy	Targeting Strategy	Cancer Type	Key Readouts	Limitations	Translational Status	Ref.
**Small molecule-loaded EVs**	Chemotherapeutic agents (e.g., doxorubicin)	Incubation, Electroporation, Sonication, Membrane permeabilization	Passive tumor accumulation (EPR effect); ligand-mediated active targeting	Breast cancer(4T1), Glioma, Ovarian cancer	Enhanced tumor accumulation; reduced systemic toxicity (e.g., cardiotoxicity); prolonged survival in murine models	Low loading efficiency; premature drug leakage; batch-to-batch variability; challenges in large-scale GMP production	Preclinical stage	[82,83]
**siRNA-loaded EVs**	Oncogene-targeting siRNAs (e.g., KRAS, PLK1)	Electroporation, Donor cell transfection, Lipid-assisted loading	Passive accumulation; peptide-engineered active targeting (e.g., RVG)	PDAC; glioblastoma	Efficient gene silencing; tumor growth suppression; minimal off-target toxicity	siRNA instability; off-target gene effects; loading variability; manufacturing scalability constraints	Early clinical stage (Phase I)	[84,85]
**miRNA mimic-based EV therapy**	Tumor suppressor miRNA mimics (e.g., miR-34a)	Donor cell overexpression, Electroporation	Passive tumor targeting; ligand-modified surface engineering	Hepatocellular carcinoma; breast cancer; colorectal cancer	Induction of apoptosis; inhibition of EMT and metastasis; tumor growth reduction	miRNA degradation; pleiotropic gene modulation; dose standardization challenges; limited in vivo persistence	Preclinical stage	[86,87]
**mRNA-loaded EVs**	Therapeutic mRNAs (e.g., p53, suicide genes, immunostimulatory mRNAs)	Donor cell genetic engineering; RNA-binding protein–mediated cargo enrichment	Surface-engineered peptide targeting; passive tumor accumulation	Melanoma, Lung cancer	Functional protein re-expression; tumor growth inhibition; immune activation	mRNA instability; variable translation efficiency; complexity in scalable production; regulatory uncertainty	Preclinical stage	[88,89]
**Immune cell-derived EVs**	MHC-peptide complexes, Co-stimulatory molecules, Tumor antigens	Antigen pulsing of dendritic cells; genetic modification	Immune cell-mediated targeting; T cell priming in lymphoid organs	Melanoma, NSCLC	CD8^+^ T cell activation; IFN-γ secretion; tumor regression in early-phase trials	Modest efficacy as monotherapy; DC heterogeneity; complex GMP manufacturing; scalability limitations	Phase I/II clinical trials	[90,91]
**Immune checkpoint-modulating EVs**	Surface PD-L1; engineered anti-PD-L1–displaying EVs	Donor cell genetic engineering; surface protein display systems	Modulation of tumor immune microenvironment	Melanoma, Breast cancer	T cell suppression or reactivation; modulation of tumor progression	Risk of systemic immune dysregulation; dual immunological effects; regulatory complexity; safety concerns	Preclinical stage	[92,93]
**EV-based cancer vaccines**	Tumor-associated antigens; neoantigens; immunoadjuvant molecules	Antigen overexpression; surface display engineering; adjuvant incorporation	APC targeting, Lymph node accumulation	Melanoma, Prostate cancer	Antigen-specific T cell responses; tumor rejection; induction of immune memory	Limited efficacy as monotherapy; need for combination therapy; manufacturing standardization challenges; personalization complexity	Early clinical stage	[94,95]

## Data Availability

No datasets were generated or analyzed during the current study.
